# Targeting Immune-Related Biological Processes in Solid Tumors: We do Need Biomarkers

**DOI:** 10.3390/ijms20215452

**Published:** 2019-11-01

**Authors:** Fabio Pagni, Elena Guerini-Rocco, Anne Maria Schultheis, Giulia Grazia, Erika Rijavec, Michele Ghidini, Gianluca Lopez, Konstantinos Venetis, Giorgio Alberto Croci, Umberto Malapelle, Nicola Fusco

**Affiliations:** 1 Department of Medicine and Surgery, Pathology, San Gerardo Hospital, University of Milano- Bicocca, 20900 Monza, Italy; fabio.pagni@unimib.it; 2 Division of Pathology, IEO-European Institute of Oncology IRCCS, 20141 Milan, Italy; elena.guerinirocco@ieo.it; 3 Department of Oncology and Hemato-Oncology, University of Milan, 20122 Milan, Italy; 4 Department of Pathology, University Hospital of Cologne, Institute of Pathology, 50924 Cologne, Germany; anne.schultheis@uk-koeln.de; 5 Department of Research, Human Tumors Immunobiology Unit, Fondazione IRCCS Istituto Nazionale dei Tumori, 20133 Milan, Italy; Giulia.Grazia@istitutotumori.mi.it; 6 Division of Medical Oncology, Fondazione IRCCS Ca’ Granda-Ospedale Maggiore Policlinico, 20122 Milan, Italy; erika.rijavec@policlinico.mi.it (E.R.); michele.ghidini@policlinico.mi.it (M.G.); 7 School of Pathology, University of Milan, 20122 Milan, Italy; gianluca.lopez@unimi.it; 8 Pd.D. Program in Translational Medicine, University of Milan, 20122 Milan, Italy; 9 Division of Pathology, Fondazione IRCCS Ca’ Granda-Ospedale Maggiore Policlinico, 20122 Milan, Italy; giorgio.croci@unimi.it; 10 Department of Pathophysiology and Transplantation, University of Milan, 20122 Milan, Italy; 11 Department of Public Health, University of Naples Federico II, 80137 Naples, Italy; 12 Department of Biomedical, Surgical, and Dental Sciences, University of Milan, 20122 Milan, Italy

**Keywords:** immunotherapy, immunoediting, cancer, biomarkers, melanoma, lung cancer, breast cancer, head and neck cancer, gastrointestinal tract cancer, renal cell carcinoma, urothelial cancer, breast cancer

## Abstract

Immunotherapy has become the standard-of-care in many solid tumors. Despite the significant recent achievements in the diagnosis and treatment of cancer, several issues related to patients’ selection for immunotherapy remain unsolved. Multiple lines of evidence suggest that, in this setting, the vision of a single biomarker is somewhat naïve and imprecise, given that immunotherapy does not follow the rules that we have experienced in the past for targeted therapies. On the other hand, additional immune-related biomarkers that are reliable in real-life clinical practice remain to be identified. Recently, the immune-checkpoint blockade has been approved in the US irrespective of the tumor site of origin. Further histology-agnostic approvals, coupled with with tumor-specific companion diagnostics and guidelines, are expected in this field. In addition, immune-related biomarkers can also have a significant prognostic value. In this review, we provide an overview of the role of these biomarkers and their characterization in the management of lung cancer, melanoma, colorectal cancer, gastric cancer, head and neck cancer, renal cell carcinoma, urothelial cancers, and breast cancer.

## 1. Introduction

The window of opportunity for precision medicine in the management of cancer is rapidly widening in this era of immuno-oncology. Our improved understanding of the mechanisms underpinning cancer immunoediting and immunomodulation uncovered not only the prognostic value of new biomarkers but also their predictive role in determining the likelihood of clinical benefit from immunotherapy [1]. This has represented a watershed in cancer diagnosis, basic sciences investigations, and clinical trial design. To date, the naïve vision of a single-feature-based patient selection for immunotherapy has become insufficient [2]. In real-life practice, indeed, the clinical ramifications of cancer management are extremely intricate. In this review, we seek to outline the role of immune-related biomarkers in the management of solid tumors, focusing on the state-of-the-art armamentarium available for their pathological characterization.

## 2. The Three Phases of Immunoediting

A deep understanding of the mechanisms underlying the immunoediting process can provide insights into the determinants of immunotherapy efficacy [3]. For a long time, the role of the immune system in cancer control has been controversial. With the implementation of immunodeficiency in mouse models, the importance of immune cell activity in the rejection of transplanted tumor cells has been uncovered [4]. In vivo experiments have revealed that immunodeficient subjects are more tumor-prone than immunocompetent individuals [5,6,7]. Many studies indicate that immune cell activity is able to modify and even trigger the development of neoplasms. Not only their quantity, but also quality, and spatial distribution, tumor-infiltrating lymphocytes (TILs) are related to patient’s survival in several cancer types, akin the presence of IFN-g producing cells [8,9]. Further insight into the role of the immune system in tumors were provided by the observation of different degrees of spontaneous immune responses in cancer patients [10]. In this scenario, the addition of “avoiding immune destruction” by Hanahan and Weinberg in their revised version of the hallmarks of cancer is emblematic [11].

The neoplastic cell can stochastically acquire the ability to overcome intrinsic mechanisms of elimination of transformed cells by the immune system. This process, known as cancer immunoediting, consists of three sequential phases, “elimination”, “equilibrium”, and “escape” [10]. In the elimination phase, recognition of transformed cells leads to the activation of the two arms of the immune system. Specifically, dendritic cells present tumor antigens in the context of major histocompatibility complex (MHC) to prime cancer-specific T-helper CD4+ cells and recruit CD8+ cytotoxic T lymphocytes (CTL). T lymphocytes cooperate with players of the innate immunity, such as natural killer (NK) cells and macrophages, with the ultimate effect of eliminating malignant cells before the tumor becomes evident [12]. Eventually, the tumor cells may acquire additional genetic aberrations by means of selective pressure, leading to resistant neoplastic sub-clones capable of surviving to the immune surveillance [13]. During the equilibrium period, adaptive immunity represents a shelter for tumor growth. In this phase, the neoplasm is present but tumor cells are maintained in a functional state of “dormancy” [14]. The continuous activity of the immune system against cancer cells progressively sculpts the tumor’s immunogenicity: genomic instability of tumor cells can result in new clones able to avoid immune recognition and destruction [15]. During the escape phase, the neoplastic cells circumvent the immune-mediated killing and start growing unrestrained, giving rise to a clinically evident disease [16,17]. Among the immune-checkpoint pathways, those represented by programmed cell death protein 1 (PD-1) and CTL-associated protein 4 (CTLA-4) have a prominent role in the setting of immunotherapy [18]. Hence, the blockade of these immune checkpoints can reverse the mechanism of tumor-induced immunosuppression, leading to effective anti-tumor immunity [19,20,21,22,23].

Immunoediting plays a crucial role both in cancer progression and in its relapse [12]. Among the mechanisms of tumor escape from immune surveillance, the most investigated are the alterations that lead to reduced immune recognition and consequent reduced immune cell stimulation. In particular, downregulation or loss of tumor antigens, as well as mutations in the antigen-presenting machinery, emerge from genetic instability of tumor cells and result in the selection of poorly immunogenic cancer cell variants able to progressively growth becoming “invisible” to the immune system [24]. Instead, the immune escape can occur through the expression of immune-suppressive ligands, such as the programmed cell death-ligand 1 (PD-L1), as well as the recruitment of immunosuppressive populations (e.g., regulatory T cells or myeloid-derived suppressor cells) [25].

## 3. Tailoring Immunotherapy in Real-Life Clinical Practice

**Figure 1:**
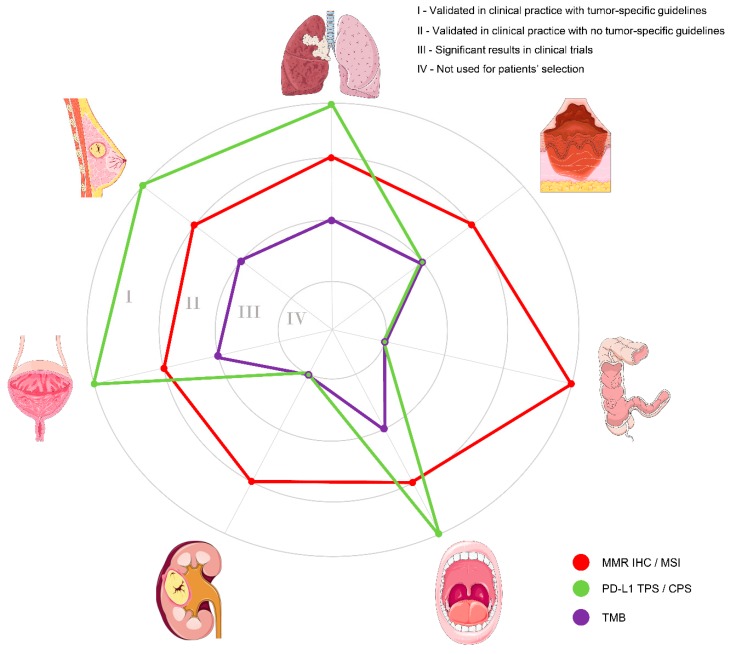
Schematic representation of the main tools available for patients’ selection for immunotherapy, according to the different anatomical sites. Clockwise from the top: non-small cell lung cancer, cutaneous melanoma, colorectal cancer, head and neck squamous cell carcinoma, renal cell carcinoma, urothelial carcinoma, and breast cancer. Each circle represents a level of validation of the various tools, as reported on the top right. The diagnostic tests are color-coded on the basis of the legend on the bottom right. MMR, mismatch repair; IHC, immunohistochemistry; MSI, microsatellite instability; PD-L1, programmed cell death ligand 1; TPS, tumor proportion score; CPS, combined positive score; TMB, tumor mutational burden.

After the histology-agnostic approval of pembrolizumab in mismatch repair (MMR)-deficient tumors, the predictive molecular pathology scenario in cancer immunotherapy changed radically [26]. For the first time in the history of medicine, a drug (in this case, an immune-checkpoint inhibitor) was approved based on a specific molecular feature of the neoplasm, irrespective of its anatomical site of origin. A multitude of different tools has been proposed to inform immunotherapy, including morphology, immunohistochemistry (IHC), polymerase chain reaction (PCR)-based techniques and next-generation technology assays, such as next-generation sequencing (NGS) or multiplex barcode technology (e.g., NanoString) [27]. However, not all the available tools are validated for patients’ selection in clinical practice, as shown in Figure 1.

Among new biomarkers to select patients for immunotherapy, the tumor mutational burden (TMB) has been shown a strong correlation with the response to some compounds [28]. TMB is defined by the total number of somatic nonsynonymous mutations per coding area of the tumor DNA. It has been hypothesized that tumors with a higher TMB are more likely to express neoantigens and to induce a more robust immune response in the presence of immune checkpoint inhibitors [28]. Regrettably, the TMB analysis is considered expensive, time-consuming, and deceptive if the analyses are carried out with an unsuitable NGS panel [5,6]. Another important facet in TMB analysis is represented by the lack of widely adopted guidelines and recommendations for its assessment and reporting. Initially, TMB was determined using wide approaches (e.g., whole exome sequencing), but more focused gene panels are currently being explored [29,30,31,32,33]. In addition, there are several indications that TMB is not a universal biomarker, as its value varies not only across tumor types but also across different genomic regions and during the time [2]. This notion highlights the need for disease-specific TMB panels and thresholds.

**Figure 2:**
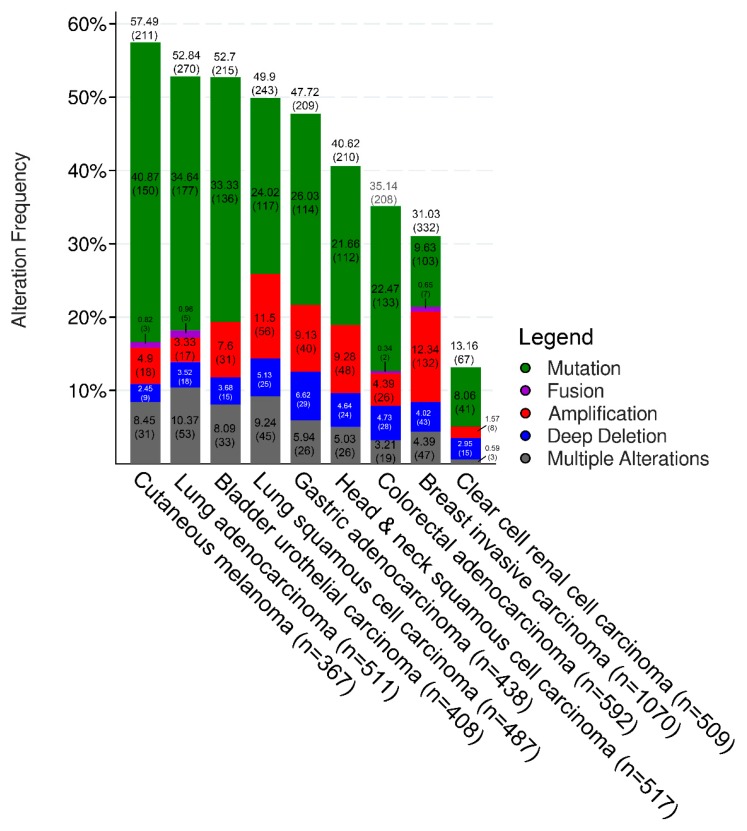
Mutation frequency in selected immune- and DNA damage response-related genes across different tumor types. The TCGA PanCancer Atlas available at cBioPortal.com was investigated for molecular alterations (color-coded on the basis of the legend on the right) targeting *CHEK1, CHEK2, RAD51, BRCA1, BRCA2, MLH1, MSH2, ATM, ATR, MDC1, PARP1, FANCF, HLA-A, HLA-B, HLA-C, HLA-E, HLA-F, HLA-G, HLA-K, HLA-L, SERPINB3, SERPINB4, JAK2, B2M,* and *STK11* in 4899 cases of 9 cancer types.

Likewise, the analysis of the MMR status is troubled by the vastity of the existing diagnostic methods in the substantial absence of companion diagnostic (CD) tests [27]. In general, MMR IHC is mirrored by microsatellite instability (MSI) in endometrial and colorectal cancers [34,35]. However, not all MMR-deficient tumors show MSI (e.g., breast cancers), questioning the interchangeability of these analyses as pan-cancer predictive tests [36]. Hence, MMR/MSI assays have been originally developed by geneticists to identify Lynch syndrome families and not for choosing the optimal drug to treat acquired tumors. Given the wide heterogeneity in the repertoire of molecular alterations in immune-related genes across different tumor types (Figure 2), novel, efficient, reproducible, and reliable techniques coupled with tumor-specific methods and guidelines are needed. 

Another important issue related to the patient’s selection for immunotherapy is represented by the PD-L1 analysis by IHC. In this respect, important harmonization efforts have been made to standardize both the preanalytical and interpretative phases of PD-L1 testing, at least in non-small cell lung cancer (NSCLC) [37]. The reproducibility of PD-L1 testing in real-life clinical practice evaluated both for “closed” and “open” platforms, showed overlapping results, particularly when the 22C3 antibody clone was used [37]. On the other hand, there are several clues to advise that the same interpretation guidelines should not be translated across different tumor types. For example, the tumor proportion score (TPS) works perfectly for lung cancer but not for head and neck cancer, where the combined positive score (CPS) is more reliable [38]. These two scoring systems are rather different, given that the former considers only the percentage of PD-L1-positive neoplastic cells, while the latter combines all PD-L1-positive cells (i.e., tumor cells, lymphocytes, and macrophages) into the following formula.

CPS=NumberofPDL1positivecellsTotalnumberofviabletumorcells × 100

Although the CPS can theoretically exceed the value of 100, the maximum score is defined as 100. Fascinating perspectives are being provided by experimental models addressing the plasticity of the cellular inflammatory response of the host. There are several lines of evidence to suggest that the myeloid-derived cell function is a finely tuned mechanism to control tumor growth. Specifically, both in local and hematopoietic niches, its crosstalk with the tumor cells as well as with exogenous stimuli, is based on the microbiota repertoire [39,40].

## 4. Immune-Related Biomarkers in Solid Tumors: Prognostic and Predictive Role

### 4.1. Non-Small Cell Lung Cancer

The rulemaking biomarker in NSCLC immunotherapy prediction is represented by PD-L1 [41]. The membrane expression of this protein is evaluated using the TPS by IHC [37]. To date, pembrolizumab, an anti-PD-1 drug, is a standard first-line treatment in advanced NSCLC with high PD-L1 TPS (i.e., positivity in at least ≥50% of tumor cells by IHC in patients without sensitising alterations involving the epidermal growth factor receptor (*EGFR*) or anaplastic lymphoma kinase *(ALK*) genes) [23]. However, not all immune-checkpoint inhibitors show the same performance. Based on the insights provided by recent randomised phase 3 trials, while pembrolizumab monotherapy can be extended as first-line therapy to patients with locally advanced or metastatic NSCLC in patients with low PD-L1 expression (i.e., TPS ≥ 1%) [42], negative findings were achieved for nivolumab (an anti-PD-L1 drug) alone (CheckMate-026) or in combination (MYSTIC trial) [43]. It should be noted, however, that the degree of heterogeneity in the results of clinical trials for immunotherapy in NSCLC is remarkable. Hence, the combination of pembrolizumab with platinum-based chemotherapy in advanced squamous NSCLC and nonsquamous NSCLC has been considered beneficial, regardless of the PD-L1 TPS [44,45]. On the other hand, PD-L1, albeit imperfect, remains an important biomarker to select patients for immunotherapy. Among other possible predictive biomarkers, the T- effector, and interferon-𝛾 gene signature was related to improved OS [46]. Immune-checkpoints blockade in combination with tyrosine kinase inhibitors (TKIs) have also been explored. Regrettably, this association is characterized by very high toxicities [47,48]. For this reason, the role of immunotherapy in patients with mutated *EGFR* or rearranged *ALK* NSCLC remains controversial.

### 4.2. Melanoma

Similarly to NSCLC, the presence of TILs, and in particular CD8+ T cells, is associated with favorable outcome in melanoma patients [49,50]. However, the prognostic role of TILs testing in melanoma remains a matter of controversy [51,52]. Recently, Fu et al. performed a systematic review and meta-analysis that demonstrated the favorable prognostic role of CD3+, CD4+, CD8+, FOXP3+, and CD20+ TILs in melanoma patients [53]. Additionally, a study conducted by Tumeh et al., showed that in patients affected by metastatic melanoma treated with pembrolizumab, the responders had a higher number of CD8+ T cells, associated with higher PD-1/PD-L1 expression, at the the invasive front of the tumor [54]. Results of trials investigating the prognostic role of PD-L1 expression in melanoma are discordant [55,56,57]. Although PD-L1 is the most investigated biomarker, to date there is not a consensus regarding its predictive role to the outcome for immunotherapy in melanoma. A correlation between PD-L1 expression and response to immunotherapy in patients affected by metastatic melanoma has been reported in several studies [58,59]. The CheckMate 067 trial revealed some intriguing results. In this phase 3 study of nivolumab (or nivolumab plus ipilimumab) versus ipilimumab alone in previously untreated advanced melanoma, immunotherapy led to an overall survival (OS) benefit in patients with a lower tumor PD-L1 expression level. No difference in terms of OS has been achieved between the nivolumab-plus-ipilimumab arm and the nivolumab arm among patients with a tumor PD-L1 expression ≥1% or ≥5% [60]. However, durable responses with anti-PD-1 therapy have also been reported in patients with PD-L1 negative tumors [61,62].

To date, several issues remain to be accurately defined, such as the assay to determine PD-L1 expression and the cut-off values for the definition of PD-L1 positivity. Lymphocyte activation gene-3 (LAG-3) is an immune checkpoint, expressed by TILs, able to suppress T cell activation and cytokines release [63]. It is currently under investigation whether LAG-3 could be used as a predictive biomarker for immunotherapy. Recently, the count of eosinophils granulocyes has been proposed as a bona fide predictive biomarker for immunotherapy response in melanoma patients [64]. Hence, an increase in eosinophils has been observed in patients treated with ipilimumab or pembrolizumab showing a better OS [65,66]. Moreira et al. showed the prognostic role of the eosinophilia in 177 melanoma patients. A trend toward longer survival has been observed in patients that have experienced an increase in eosinophil count regardless of the treatment received [67]. Recently, a prognostic role of eosinophil cationic protein (ECP), a protein released by degranulation of eosinophils, has been reported in melanoma patients. Patients with higher ECP serum levels showed a shorter survival compared to patients with lower ECP serum levels, regardless of the therapy received and of presence of eosinophilia [68].

### 4.3. Gastrointestinal Tract Cancers

In contrast to other tumor types, immunotherapy has not yet become a standard of treatment in gastrointestinal cancers. In colorectal cancer (CRC), four different molecular subtypes were identified as prognostic and predictive tools [69]. The consensus molecular subtype 1 (CMS1) has a prevalence of 14% in CRCs and showed high levels of MSI, being assessed as MSI-high (MSI-H) [70]. In refractory unresectable or metastatic MSI-H or MMR-deficient CRCs (4% of all metastatic CRC), U.S. Food and Drug Administration (FDA) approved the use of the anti-PD-1 agent pembrolizumab and nivolumab [71]. However, even in MSI-H patients, the objective response rate to checkpoint inhibitors has been unsatisfactory, with values between 31.1 and 40% [72,73]. For this reason, together with MSI-H, the evaluation of elevated microsatellite alterations at selected tetranucleotide repeats (EMAST) has been recently proposed. Clinical features and molecular profiles of MSI-H and EMAST-positive CRCs were similar (51.3%). However, EMAST-positive and MSI-H CRCs had longer survival compared to EMAST-negative and MSI-H tumors (*p* < 0.001). A higher TMB was detected in EMAST-positive and MSI-H CRCs compared to EMAST-positive or MSI-H disease alone. Therefore, the combination of MSI-H and EMAST-positive CRCs with high TMB may identify the best patients to a candidate for immunotherapy [34]. The gut microbiome plays an important role in response to immunotherapy, as well [40]. Different bacterial strains may inhibit or enhance PD-L1, acting differently on T-cells and on CTLA-4 blockade. For instance, Fusobacterium nucleatum has been correlated with poor outcome during immunotherapy by activating interleukins as inflammatory mediators, inhibiting both NK cells and TILs [74].

In gastric cancer (GC), four different molecular subtypes were identified [75,76]. MSI tumors, accounting for 22% of GCs, harbor high TMB and increased number of neoantigens [76]. In parallel, EBV-positive tumors (10% of GC) are generally enriched in cytotoxic T lymphocytes and have a high rate of response to anti-PD-1 and anti-PD-L1 inhibitors [77]. Recent results in patients treated with pembrolizumab in advanced and refractory GC showed a higher objective response rate (ORR) in PD-L1 positive tumors (15.5 vs. 6.4% for PD-L1 negative). However, patients with PD-L1 negative disease experienced responses as well, including three complete responses (2.8%) [78]. In resectable GC, low PD-1 and PD-L1 mRNA peripheral blood levels were reported to be independent poor prognostic predictors for OS [79]. In the same setting, MSI status has a strong prognostic and predictive role. Indeed, in a meta-analysis of four trials of resected patients, MSI-H patients had longer 5-year overall survival (OS, 77.5 vs. 59.3%) and disease-free survival (DFS, 71.8 vs. 52.3%) with respect to microsatellite stable (MSS) patients. Moreover, MSI-H patients did not benefit from preoperative chemotherapy while MSS cases did (OS 62 vs. 53% for surgery alone, DFS 57 vs. 41%). Thus, MSI status should be considered in case neoadjuvant or adjuvant CT is administered. MSI-H patients do not benefit from CT and may be good candidates either to surgery upfront or immunotherapy [80].

### 4.4. Head and Neck Cancer

Patients with recurrent or metastatic head and neck squamous-cell carcinoma (HNSCC) after platinum chemotherapy have a dramatically poor prognosis and limited therapeutic options [81]. The immune-checkpoint blockade has only recently been demonstrated to be beneficial in these patients [38]. The rational trait underpinning of immunotherapy in these tumors is represented by several mechanisms. These include impairment of tumor-infiltrating T lymphocytes and of NK cell activity, poor antigen-presenting function, accumulation of tumor-secreted proteins that inhibit stimuli, T-cell apoptosis, and presence of T-regulatory cells that repress T cells induction and proliferation [82]. Recently, both treatments with nivolumab [83] and with pembrolizumab [84] resulted in longer OS compared to the standard, single-agent therapy in patients with platinum-refractory, recurrent or metastatic HNSCC. These observations were irrespective of PD-L1 expression or human papillomavirus (HPV) status. Based on these data, immunotherapy with these two agents has been approved by the FDA for the second-line treatment of recurrent and/or metastatic HNSCC [85]. Lately, the opportunity for testing PD-L1 in these tumors has become a subject of controversy among pathologists and oncologists. Hence, the survival benefit in tumors showing PD-L1 TPS ≥ 1% is greater compared to that in unselected HNSCC treated with nivolumab [83]. Several data indicate that, in HNSCC, PD-L1 is expressed by both tumor and tumor-associated immune cells, and it is significantly associated with the clinical outcome [86]. For this reason, the CPS is recommended in these tumors to select patients for immune checkpoint blockade. Hence, in HNSCC patients with CPS >20, OS is significantly longer with pembrolizumab than standard treatment.

Nasopharyngeal carcinoma (NPC) has been reported to be highly associated with Epstein-Barr virus (EBV) showing multifactorial etiology and specific geographic distribution [87,88]. The EBV expresses proteins that contribute to tumour progression and immune escape, including LMP1, LMP2 and EBNA1 [89]. In particular, LMP1 in collaboration with INF-γ regulates the expression of PD-L1 [90,91]. Although these proteins have not been studied as potential biomarkers, they may represent promising targets for immunotherapy since they highly interact with the immune system. Among the available treatment options for NPC patients, radiotherapy represent a cornerstone [92]. Based on the tumor stage, radiotherapy can be used either alone or in combination with chemotherapy [93,94]. Recently, immunotherapy showed important results against NPC, mostly by using immune checkpoint blockade [95,96]. Interestingly, EBV-positive NPC are usually characterized by dense infiltration of lymphocytes in the tumor stroma and positive PD-L1 expression in tumor cells, resulting in extremely attracting targets for immune-checkpoint inhibitors [97]. Recent randomized clinical studies have examined the antitumor activity of pembrolizumab and nivolumab in patients with recurrent or metastatic NPC. The latter was tested in 44 patients showing 20.5% ORR while pembrolizumab in 27 patients demonstrated almost 26% ORR. Moreover, both drugs presented higher efficacy in patients with PD-L1 positive (>1% expression) tumors compared to those with PD-L1 negative tumors [98,99]. Other potential predictive biomarkers such as tumor-associated macrophages and regulatory T cells (e.g., FOXP3, HLA-A and HLA-B) have been proposed [98,100]. However, further investigation is necessary.

### 4.5. Renal Cell Carcinoma

During the past decades, the field of renal cell carcinoma (RCC) has experienced a paradigm shift in the clinical management, particularly for metastatic settings. Current guidelines recommend therapies with agents targeting vascular endothelial growth factor (VEGF) (e.g., sunitinib, sorafenib, pazopanib, bevacizumab and axitinib) and mammalian target of rapamycin (mTOR) pathways (e.g., temsirolimus and everolimus) as first-line treatment, while non-specific cytokines are suggested as an alternative option for selected patients [101]. Based on superiority in terms of objective response rate (ORR) and OS compared to everolimus, nivolumab was the first immune checkpoint inhibitor that granted approval in advanced RCC, as second-line therapy [102,103]. To date, both Checkpoint inhibitors and TKI are valid second-line strategies in metastatic RCC. These two families of drugs are characterized by totally different mechanisms of action that have not been really compared head-to-head in randomized clinical trials [104]. Furthermore, one of the most critical issues in patients’ selection is represented by the substantial lack of robust predictive biomarkers. As a consequence, these patients are managed using vastly heterogeneous clinical schemes. These include best timing and optimum sequence, mode of action, response to first-line TKI treatment, individual patient-related factors, and treatment-related factors (e.g., toxicities) [104].

### 4.6. Urothelial Cancer

Urothelial carcinoma (UC) represents a prototypical immunogenic tumor. A variety of inflammatory cells reportedly infiltrates UC, including CD8+ TILs, tumor-infiltrating neutrophils and CD68+ tumor-associated macrophages [105,106,107]. The very first immunotherapeutic agent (i.e., Mycobacterium bovis bacillus Calmette-Guerin), which recruits immune cells to target UC tumor cells when instilled locally, has been used for the treatment of non-muscle-invasive UC for nearly four decades [108]. An analysis of publicly available data on the online database cBioPortal showed a high frequency of mutations in DNA damage response genes (i.e., *CHEK1, CHEK2, RAD51, BRCA1, BRCA2, MLH1, MSH2, ATM, ATR, MDC1, PARP1, FANCF*) in 1249 UC cases from 8 different studies (range 38.1–5.9%), as shown in Figure 2 [109]. In addition, a substantial proportion of UCs express PD-L1, both in tumoral cells (20%) [110] and in tumor-infiltrating inflammatory cells (40%) [111]. Not surprisingly, PD-L1 expression correlates with poor prognosis in these tumors [110,111]. The potential predictive role of PD-L1 expression in relation to treatment with immune checkpoint inhibitors has been studied in clinical trials with nivolumab, durvalumab, avelumab, atezolizumab and pembrolizumab [112,113,114,115,116,117,118,119,120]. A strong association between PD-L1 expression and the overall response was noted only for durvalumab [121,122], using the assay Ventana SP263 (which is also an FDA-approved companion diagnostic in UC), positive staining in either ≥25% of tumor or immune cells is predictive of response [123]. Other clinical trials with nivolumab, avelumab, atezolizumab, and pembrolizumab, which utilized different assays (Dako 22C3 and 28-8, Ventana SP26 and SP142), failed to demonstrate a strong correlation between PD-L1 expression and response, inter-assay variability, as well as the use of different compartment scorings and cut-offs, are believed to contribute to the lack of predictive correlation in these studies [120].

From a molecular point of view, UC generally harbors a high mutational load [124]. The overall TMB represents another promising biomarker with predictive value in UC. It has been recently shown that responders to atezolizumab in UC expressed a higher median mutational load than nonresponders (12.4 mutations/Mb vs. 6.4 mutations/Mb) [116]. Other surrogate biomarkers of TMB, such as a deficit in the MMR system, MSI and polymerase-ε (*POLE*) mutations have been studied in other types of cancers [72,125] and could potentially be useful in UC.

Compared to PD-L1 IHC and TMB, the immune cell gene expression profiling is considered a more comprehensive immune biomarker by many Authors. This integrative analysis allows for the assessment of the tumor microenvironment and its inflammatory status [9]. Several panels with genes involved in T cell signaling, antigen presentation, and additional immunomodulatory functions have been investigated in different malignancies, in relation to a possible predictive role for ICI response [120,126]. An interferon-gamma 25-gene signature was recently adopted to evaluate the immune microenvironment status in relation to response to treatment with nivolumab [112]. Higher values in the *IFN-γ* gene signature were correlated with response to treatment (33.9% with high IFN-γ signature vs.16.1% with non-high *IFN-γ* signature). In addition, patients with high expression of CXCL9 and CXCL10 in the same study correlated with higher response rates.

### 4.7. Breast Cancer

Breast cancer has been traditionally regarded as a weakly immunogenic neoplasm [127]. For this reason, it remains one of the tumors that have not yet experienced the bang of immunotherapy. Among breast cancer subtypes, triple-negative breast cancer (TNBC) is characterized by greater stromal and intratumoral TILs, higher PD-L1 expression and TMB, features that suggest a potential role for immunotherapy [128]. However, TNBCs are extremely heterogeneous and encompass a constellation of tumor types with different morphology, molecular features, immunogenicity, and responses to therapies [129,130,131]. There is evidence that pembrolizumab may have actual and durable antitumor activity as a single agent in a small subset of metastatic TNBC, with a higher probability of success in earlier lines of treatment and in PD-L1-positive patients [132]. It has recently been observed that PD-L1, T-cell–inflamed gene-expression profile, and TMB analyses, separately or in combination, could be useful in immunotherapy prediction also in estrogen receptor (ER)+ breast cancers [133]. In the large phase I KEYNOTE-028, pembrolizumab was tested in patients with ER+/HER2− tumors [128]. Avelumab, another anti-PD-L1 agent, was administered as monotherapy in heavily pretreated breast cancers, with modest results [134]. Differently from stromal TILs, intratumoral CD8 cells were associated with PD-L1 expression by both neoplastic cells and lymphocytes and show a prognostic value [134]. Several clinical trials are ongoing in early stages of breast cancer, with the first results coming from the neoadjuvant setting, particularly in high risk, HER2-negative breast cancers. In these patients, the addition of pembrolizumab to chemotherapy improves pathological complete response rates [135]. Despite these data are encouraging, no biomarkers have been identified to select effectively these patients. Further studies are needed to characterize the role of novel, old, and even surrogate biomarkers in breast cancer immunotherapy, focusing on tumor-specific guidelines, testing methods, complementary diagnostic tests.

## 5. Conclusions and Immediate Future Perspectives

In conclusion, it is not just the tumor’s genetic profile that can affect immunotherapy response. Additional criteria to stratify patients into clinically meaningful subgroups should be implemented to allow for tailored diagnostic programs. Hitherto, PD-L1 IHC, as well as TMB, remain imperfect tools for deciding the use of anti-PD-1 or anti-PD-L1 drugs. A deep characterization of the tumor microenvironment and microbiome immune state would be extremely beneficial. There are emerging tumour antigens, effector T-cell function, and immune-suppressive mechanisms that are currently been tested in substitution or as complementary tests for immune-checkpoint blockade. However, further studies aiming to address relationships between PD-1 and PD-L1 expression, TILs, T-cell subpopulations, neoantigen formation, and TMB are ongoing to creating an optimized model. Additional mechanisms of T-cell exclusion will be included in future biomarker development at a single patient level. So, where are we now? The emerging picture is that a combination of biomarkers is going to be required to predict a patient’s response to immunotherapy, providing that we will be able to move to a new holistic clinicopathologic approach. This represents a true challenge in contemporary cancer treatment.

## Abbreviations

TILs	Tumor-infiltrating lymphocytes
MHC	Major histocompatibility complex
CTL	Cytotoxic T lymphocytes
NK	Natural killer
PD-1	Programmed cell death-protein 1
CRLA-4	CTL-associated protein 4
PD-L1	Programmed cell death-ligand 1
MMR	Mismatch repair
IHC	Immunohistochemistry
PCR	Polymerase chain reaction
NGS	Next-generation sequencing
TMB	Tumor mutational burden
CD	Companion diagnostic
MSI	Microsatellite instability
NSCLC	Non-small cell lung cancer
TPS	Tumor proportion score
CPS	Combined positive score
EGFR	Epidermal growth factor receptor
TKIs	Tyrosine kinase inhibitors
LAG-3	Lymphocyte activation gene-3
ECP	Eosinophil cationic protein
CRC	Colorectal cancer
MSI-H	Microsatellite instability high
FDA	Food and Drug Administration
GC	Gastric cancer
OS	Overall survival
DFS	Disease-free survival
MSS	Microsatellite stable
HNSCC	Squamous-cell carcinoma of the head and neck
ORR	Objective response rate
HPV	Human papillomavirus
UC	Urothelial carcinoma
POLE	polymerase-ε
TCGA	The Cancer Genome Atlas
TNBC	Triple-negative breast cancer
ER	Estrogen receptor
